# Genome Sequence of a Unique t2247-ST692-III Livestock-Associated Methicillin-Resistant *Staphylococcus aureus* Strain from Chicken Carcass

**DOI:** 10.1128/genomeA.00026-16

**Published:** 2016-02-25

**Authors:** Dong Chan Moon, Byung-Yong Kim, Migma Dorji Tamang, Hyang-Mi Nam, Geum-Chan Jang, Suk-Chan Jung, Hee-Soo Lee, Yong-Ho Park, Suk-Kyung Lim

**Affiliations:** a Bacterial Disease Division, Animal and Plant Quarantine Agency, Anyang-si, Gyeonggi-do, Republic of Korea; b ChunLab Inc., Seoul National University, Gwanak-gu, Seoul, Republic of Korea; c Department of Microbiology, College of Veterinary Medicine, Seoul National University, Gwanak-gu, Seoul, Republic of Korea

## Abstract

We report the draft genome sequence of a novel livestock-associated t2247-ST692-III methicillin-resistant *Staphylococcus aureus* strain designated K12S0375, which was isolated from a chicken carcass in South Korea. The K12S0375 strain contains uncommon genes, including antimicrobial resistance genes (*tetL* and *tetS*) and leukotoxin (*lukED*), and the genomic distance indicates a single lineage in a genome-based phylogenetic tree compared with 459 *S. aureus* genome sequences. This genome sequence will contribute to understanding epidemiological and genomic features of the ST692 lineage, including antimicrobial resistance and virulence genes.

## GENOME ANNOUNCEMENT

Methicillin-resistant *Staphylococcus aureus* (MRSA) is a common pathogen in humans and was recently reported in nonhumans. Furthermore, livestock-associated MRSA (LA-MRSA), mainly associated with sequence type (ST) 398, is an emerging cause of human infections (1, 2). MRSA ST692 strains were isolated from chicken carcasses and chicken slaughterhouse workers only in South Korea (3). Notably, all MRSA isolates from chicken carcasses were ST692 and increased in frequency from 0.4% in 2011 to 2.3% in 2012 (3). Although new LA-MRSA types can be transmitted to humans from food animals and animal products (3), there is limited genomic and epidemiological information about ST692 MRSA and other related types. Therefore, we investigated the molecular characterization and lineage group of an ST692 MRSA strain based on whole-genome sequencing.

A t2247-ST692-III MRSA strain designated K12S0375 was isolated from a chicken carcass in Jeonnam province of South Korea in 2012. The draft genome sequence of strain K12S0375 was obtained by Illumina MiSeq (300-bp paired-end) and Roche 454 FLX (8-kb-insert paired-end) sequencing. Short contigs (<500 bp) were excluded from the hybrid result file, and Glimmer 3 was used for gene prediction (4). Annotation was performed by homology search based on the Clusters of Orthologous Groups (COG) (5). Screening of antibiotic resistance and virulence genes was performed using the Center for Genomic Epidemiology (http://www.genomicepidemiology.org). In total, 459 genome sequences were obtained from *S. aureus* subsp. *aureus* in the EzGenome database (http://ezgenome.ezbiocloud.net) and compared with the K12S0375 strain genome using average nucleotide identity (ANI) values (6). The query genome was divided into 1,020-bp fragments, and high-scoring pairs were compared with two sequences by BLAST (7).

Here, 31 contigs (*N*_50_ = 148,340 bp) were generated using a hybrid assembly of reads from Illumina (6,413,077 reads of 150-bp paired-end; >350-fold coverage) and Roche 454 (240,863 reads of 8-kb-insert paired-end; >14-fold coverage) systems. The genome of K12S0375 was 2,889,103 bp with a total coverage of 506.04× and 32.72% GC content. The genome comprised 2,085 predicted protein-coding sequences, 56 tRNA genes, and 8 rRNA genes, and two plasmid (*repUS12*, *repUS5*) replicons were observed. For the COG distribution, 273 general function open reading frames (ORFs), 244 function unknown ORFs, and 216 amino acid transport and metabolism ORFs were abundant categories (>10% of total COG-matched counts).

Strain K12S0375 contained various antibiotic resistance genes such as aminoglycosides (*aadE*), fluoroquinolones (*norA*), and macrolides (*ermB*), as well as a combination of tetracyclines such as *tet(38)-tetL-tetS*. Diverse virulence genes were detected in strain K12S0375, including adhesions, toxins, and exoenzymes such as *leukotoxin ED*, which plays an important role in bacteremia-associated lethality in murine models by killing macrophages, dendritic cells, and neutrophils (8). Moreover, strain K12S0375 showed a monophyletic lineage in the genome tree of 459 *S. aureus* strains from the EzGenome database (http://ezgenome.ezbiocloud.net) by comparison of ANI values.

This is the first reported genome sequence for an ST692 MRSA strain. The study is also expected to promote the prevention of MRSA by providing information about antibiotic resistance and virulence.

### Nucleotide sequence accession number.

The draft genome sequence of *Staphylococcus aureus* strain K12S0375 (ST692) was deposited at GenBank under the accession number JYGF00000000.
